# Supplementary material for: SARS-CoV-2 detection is independent of microbiome composition on surfaces in a major Ontario hospital
Source: PLoS One. 2025 Dec 4;20(12):e0326403. doi: 10.1371/journal.pone.0326403 (PMC12677581; doi:10.1371/journal.pone.0326403)
Supplement: S1 Text — (PDF) [file pone.0326403.s001.pdf]

SARS-CoV-2 detection is independent of microbiome composition on surfaces in a major Ontario hospital

Nikhil George, Lauren Bradford, Aaron Hinz, Marita El Kadi, Lydia Xing, Evgueni Doukhanine, Derek R. MacFadden, Caroline Nott, Michael Fralick, Rees Kassen, Alex Wong & Laura A. Hug*

* corresponding author – laura.hug@uwaterloo.ca

Supplemental Materials

Supplemental Tables A-D p. 2-5
Supplemental Figures A-M p. 6-18

Supplemental Table A: Sample metadata for the sites selected. Sample IDs follow the convention UNIT_WEEK_SOURCE. Read numbers are post-processed reads, following removal of human-associated sequences.

Sample ID	Hospital	Unit	Source	Week	SARS-COV-2	Extraction Date	Read # (millions)
ctrl	ctrl	ctrl	ctrl	ctrl	NEG	ctrl	9.64
Main_ELE_2	A	Main Elevators	Elevator	2	POS	2020.10.15	475.9
PG_3_ELE	A	Parking Garage	Elevator	3	POS	2020.10.22	346.0
PG_4_ELE	A	Parking Garage	Elevator	4	POS	2020.11.05	237.0
PG_5_ELE	A	Parking Garage	Elevator	5	POS	2020.11.10	319.6
W3_2_ELE	B	Ward 3	Elevator	2	POS	2020.10.15	405.3
W4_2_ELE	B	Ward 4	Elevator	2	NEG	2020.10.12	648.1
W4_3_ELE	B	Ward 4	Elevator	3	NEG	2020.10.22	701.1
W4_4_ELE	B	Ward 4	Elevator	4	NEG	2020.10.27	695.7
W4_5_ELE	B	Ward 4	Elevator	5	NEG	2020.11.05	568.1
W4_7_ELE	B	Ward 4	Elevator	7	NEG	2020.11.24	570.4
W4_8_ELE	B	Ward 4	Elevator	8	NEG	2020.11.24	565.7
W6_2_ELE	B	Ward 6	Elevator	2	POS	2020.10.12	676.4
ICU_2_Floor	B	ICU	Floor	2	POS	2020.10.12	265.6
ICU_3_Floor	B	ICU	Floor	3	POS	2020.10.22	203.3
ICU_5_Floor	B	ICU	Floor	5	POS	2020.11.05	197.3
ICU_6_Floor	B	ICU	Floor	6	POS	2020.11.10	343.7
ICU_7_Floor	B	ICU	Floor	7	POS	2020.11.24	644.8
ICU_8_Floor	B	ICU	Floor	8	POS	2020.11.24	500.2
W1_2_Floor	B	Ward 1	Floor	2	POS	2020.10.12	269.0
W1_3_Floor	B	Ward 1	Floor	3	POS	2020.10.22	256.5
W1_4_Floor	B	Ward 1	Floor	4	POS	2020.10.27	617.1
W1_5_Floor	B	Ward 1	Floor	5	POS	2020.11.10	427.0
W1_6_Floor	B	Ward 1	Floor	6	POS	2020.11.10	532.1
W1_7_Floor	B	Ward 1	Floor	7	POS	2020.11.24	174.6
W1_8_Floor	B	Ward 1	Floor	8	POS	2020.12.11	214.2
W1_9_Floor	B	Ward 1	Floor	9	POS	2020.12.15	204.8
W1_11_Floor	B	Ward 1	Floor	11	POS	2020.12.15	266.3
W4_2_Floor	B	Ward 4	Floor	2	NEG	2020.10.12	552.6
W4_3_Floor	B	Ward 4	Floor	3	NEG	2020.10.22	350.9
W4_4_Floor	B	Ward 4	Floor	4	NEG	2020.10.27	291.7
W4_5_Floor	B	Ward 4	Floor	5	NEG	2020.11.05	251.3
W4_6_Floor	B	Ward 4	Floor	6	NEG	2020.11.10	126.2
W4_7_Floor	B	Ward 4	Floor	7	NEG	2020.11.24	496.3
W4_8_Floor	B	Ward 4	Floor	8	NEG	2020.11.24	519.6
W4_9_Floor	B	Ward 4	Floor	9	NEG	2020.12.15	528.7
W4_10_Floor	B	Ward 4	Floor	10	NEG	2020.12.15	286.6
W4_11_Floor	B	Ward 4	Floor	11	NEG	2020.12.15	195.5

Supplemental Table B: Prevalence of resistance mechanisms to disinfection agents across samples, in reads per million reads

Sample	Resistance to Disinfection Agent Prevalence
ICU_2_Floor	11.4
ICU_3_Floor	24.5
ICU_5_Floor	14.3
ICU_6_Floor	9.2
ICU_7_Floor	9.9
ICU_8_Floor	26.6
ME_2_ELE	13.3
PG_3_ELE	17.3
PG_4_ELE	13.8
PG_5_ELE	10.4
W1_3_Floor	17.4
W1_4_Floor	29.4
W1_5_Floor	24.7
W1_6_Floor	16.1
W1_7_Floor	14.1
W1_8_Floor	19.1
W1_9_Floor	22.3
W1_A11_Floor	12.5
W3_2_ELE	75.4
W4_2_ELE	17.0
W4_2_Floor	21.0
W4_3_ELE	12.1
W4_3_Floor	23.0
W4_4_ELE	11.5
W4_4_Floor	26.9
W4_5_ELE	15.7
W4_5_Floor	19.1
W4_6_Floor	20.7
W4_7_ELE	18.1
W4_7_Floor	15.7
W4_8_ELE	17.8
W4_8_Floor	19.0
W4_9_Floor	16.0
W4_A10_Floor	12.7
W4_A11_Floor	21.9
W6_2_ELE	26.8

Supplemental Table C: Kruskal-Wallis rank sum test of ARG abundance across Source and Unit. SARS-CoV-2 detection status was not significant and thus not explored on a gene-by-gene basis. Significant tests (p-value < 0.01) are highlighted in grey.

	SOURCE (Elevator/Floor)		UNIT (ICU, Parking Garage, Wards)	
**ARG**	**Chi-squared**	**p-value**	**Chi-squared**	**p-value**
blaCTX-M	4.142	0.04183	9.9732	0.1258
blaKPC	5.1539	0.02319	5.8906	0.4356
blanDM-1	0.16299	0.6864	6.4466	0.3751
blaOXA	0.010135	0.9198	7.5473	0.2732
blaVIM	0.41667	0.5186	6.2281	0.3981
mecA	1.6261	0.2022	13.331	0.03806
mecC	0.076572	0.782	10.545	0.1035
vanA	18.453	0.00001742	12.503	0.05164
vanB	4.1604	0.04138	1.7653	0.94

Supplemental Table D: p-values from a pairwise Wilcoxon rank sum test using a Benjamini-Hochberg correction for multiple tests which identifies four groups within the ARGs of interest. Groups and their associated non-significant p-values (> 0.01) are shaded by differing greys.

	**blaCTX-M**	**blaKPC**	**blaNDM-1**	**blaOXA**	**blaVIM**	**mecA**	**mecC**	**vanA**
**blaKPC**	0.498	-	-	-	-	-	-	-
**blaNDM-1**	$2.87x10^{-5}$	0.00159	-	-	-	-	-	-
**blaOXA**	$1.02x10^{-11}$	$8.50x10^{-14}$	$3.6x10^{-12}$	-	-	-	-	-
**blaVIM**	$2.83x10^{-5}$	0.00125	0.715	$3.4x10^{-12}$	-	-	-	-
**mecA**	$3.20x10^{-11}$	$1.48x10^{-11}$	$3.6x10^{-12}$	0.715	$3.4x10^{-12}$	-	-	-
**mecC**	$9.41x10^{-6}$	0.00017	0.169	$3.4x10^{-12}$	0.353	$3.4x10^{-12}$	-	-
**vanA**	$1.48x10^{-4}$	$1.32\ x10^{-5}$	$1.56x10^{-10}$	$8.26x10^{-6}$	$1.57\ x10^{-7}$	$5.60x10^{-6}$	$7.35x10^{-11}$	-
**vanB**	$2.83\ x10^{-5}$	0.00059	0.715	$3.4x10^{-12}$	0.912	$3.4x10^{-12}$	0.433	$1.57\ x10^{-10}$

Supplemental Figures

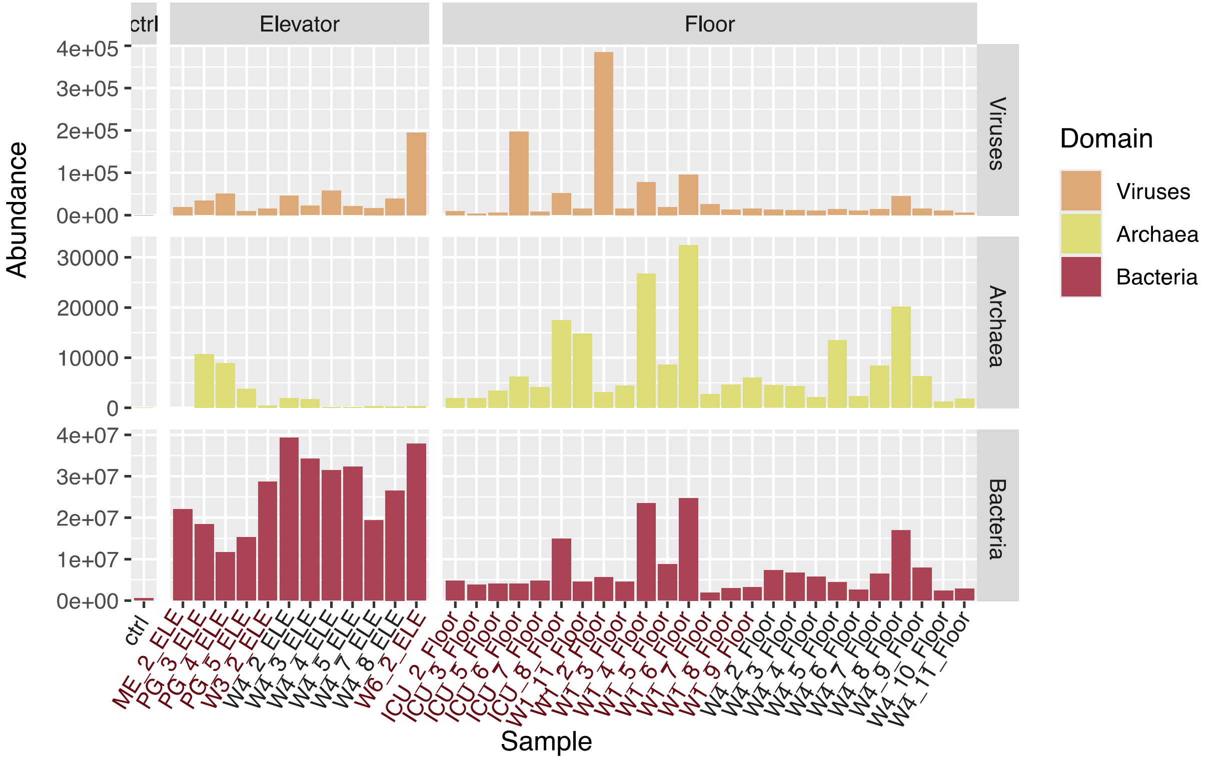

Supplemental Figure A: Total read count of Eukarya-free datasets, across Bacteria, Archaea, and viruses. Note the use of different y-axis scales to allow visualization of the lower-abundance Archaea and viruses. Sample names follow the convention UNIT_WEEK_SOURCE. SARS-CoV-2 positive samples are colored red.

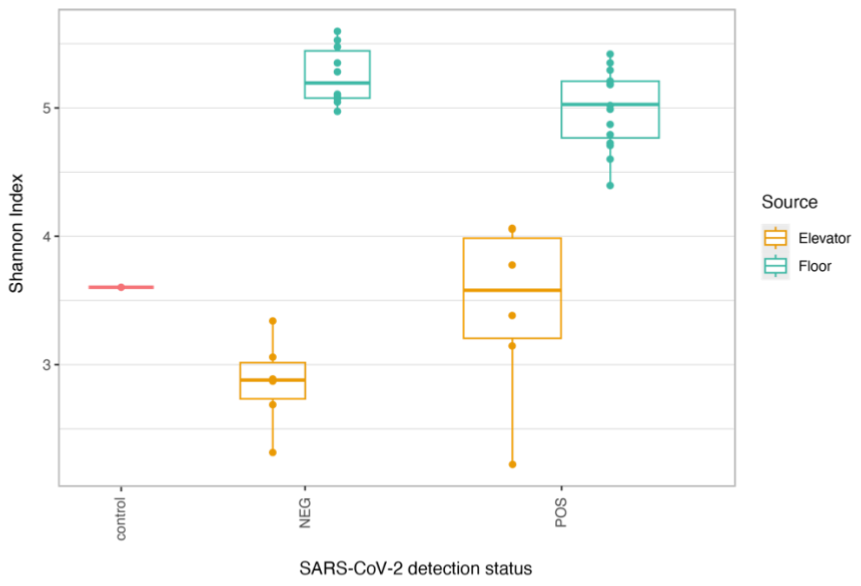

Supplemental Figure B: Shannon diversity indices for all samples, clustered by SARS-CoV-2 detection status and by site type. Box plots indicate the first and third quartile of results with the thick horizontal bar indicating the median. Whiskers identify the range of the data, with individual data points plotted. For sample Shannon diversity metrics across Units, see Supplemental Figure C.

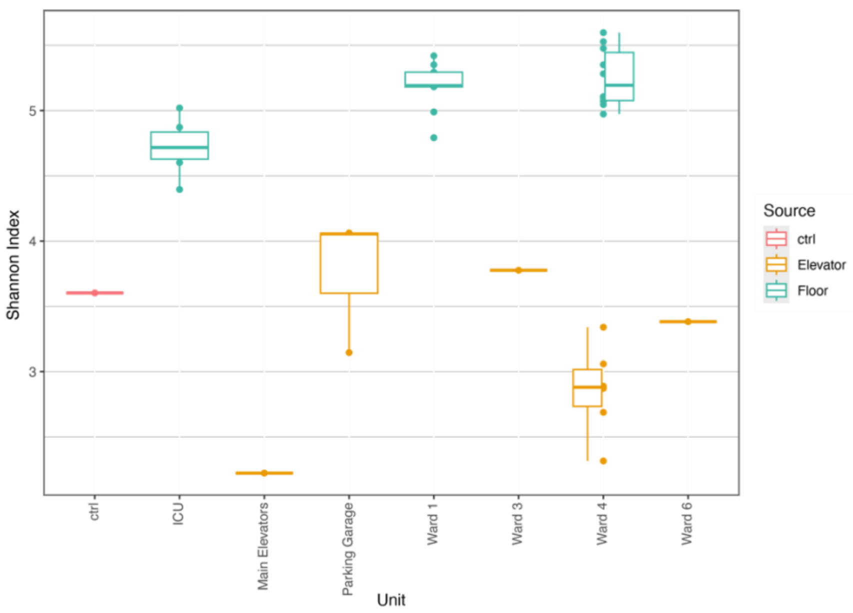

Supplemental Figure C: Shannon diversity indices for all samples, clustered by Unit and colored by Source. Box plots indicate the first and third quartile of results with the thick horizontal bar indicating the median. Whiskers identify the range of the data, with individual data points plotted.

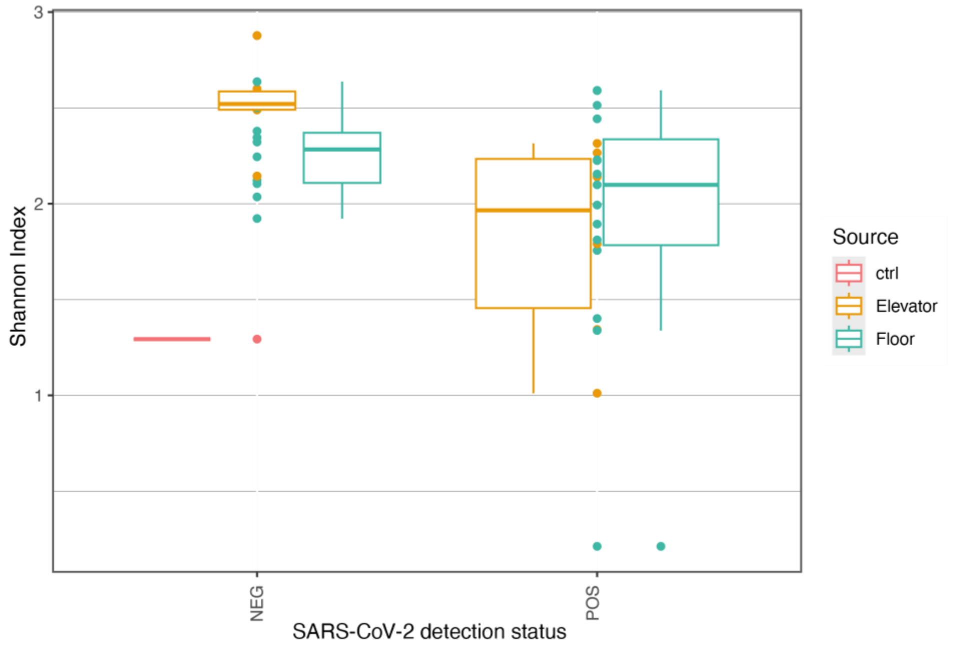

Supplemental Figure D: Shannon diversity indices for viral fraction of all samples, clustered by SARS-CoV-2 detection status and colored by Source. Box plots indicate the first and third quartile of results with the thick horizontal bar indicating the median. Whiskers identify the range of the data, with individual data points plotted.

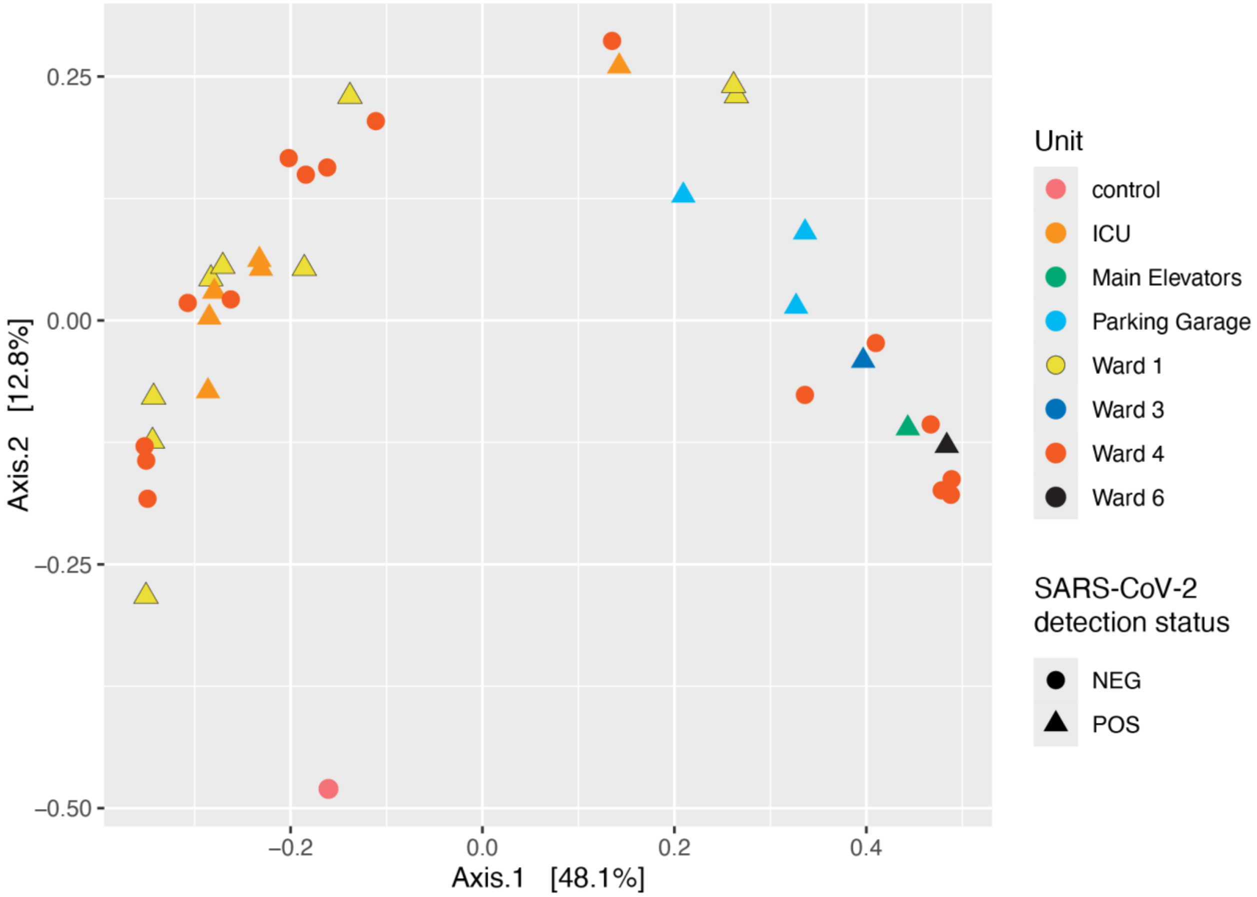

Supplemental Figure E: PCoA plot based on total microbial community composition, as in Figure 3A, with points colored by location and with SARS-CoV-2 detection indicated by shape.

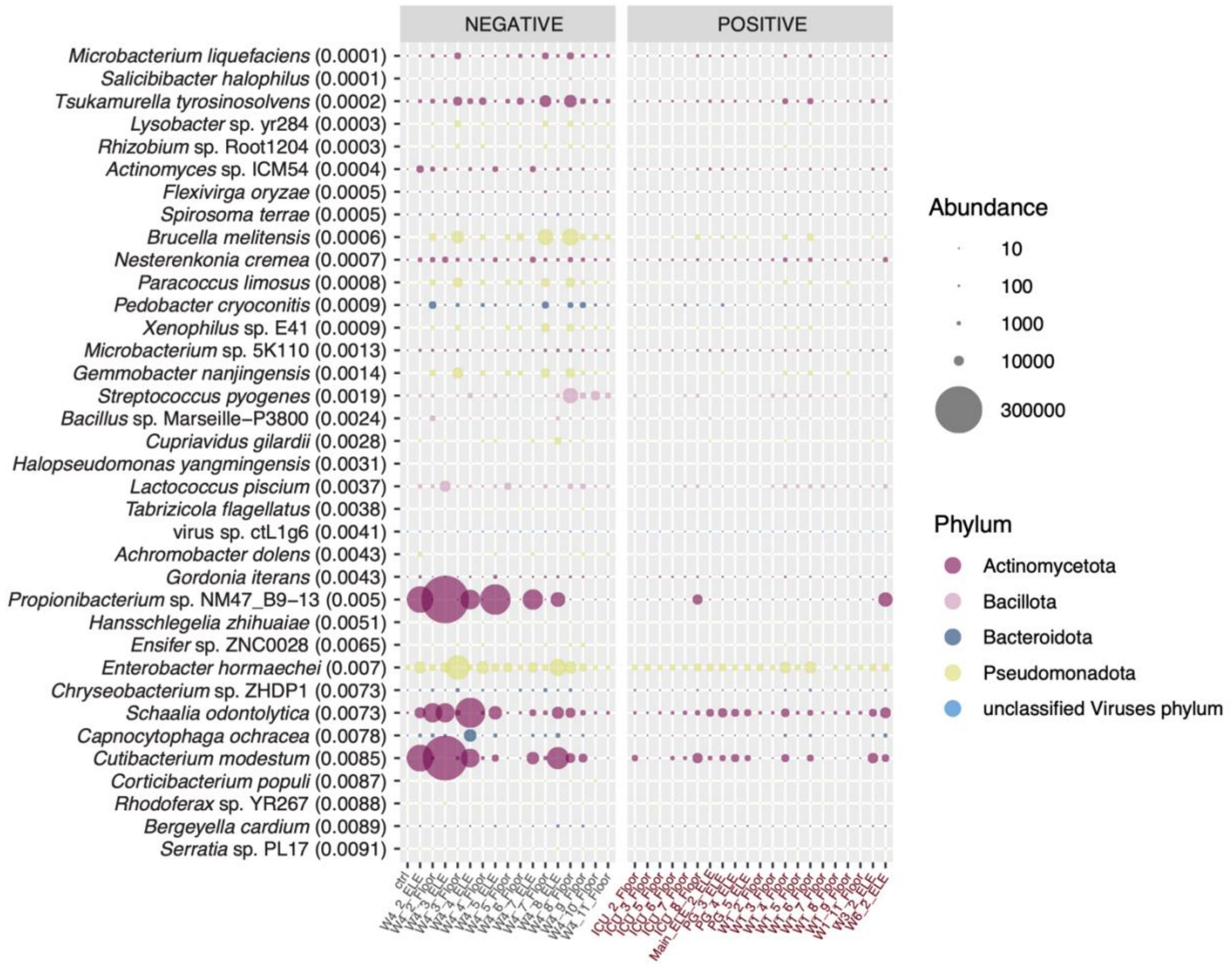

Supplemental Figure F: Abundance profiles across samples for organisms identified as significantly associated (p-value < 0.01) with negative SARS-CoV-2 detection status by indicator species analysis. Facets represent samples that were negative or positive for SARS-CoV-2 detection. Bubbles are scaled by abundance as normalized read counts and colored by phylum. Organisms are ranked by significance, with their individual p-value (p < 0.01) from the indicator species analysis noted after each organism's name in brackets. Sample names follow the convention UNIT_WEEK_SOURCE. SARS-CoV-2 positive samples are colored red.

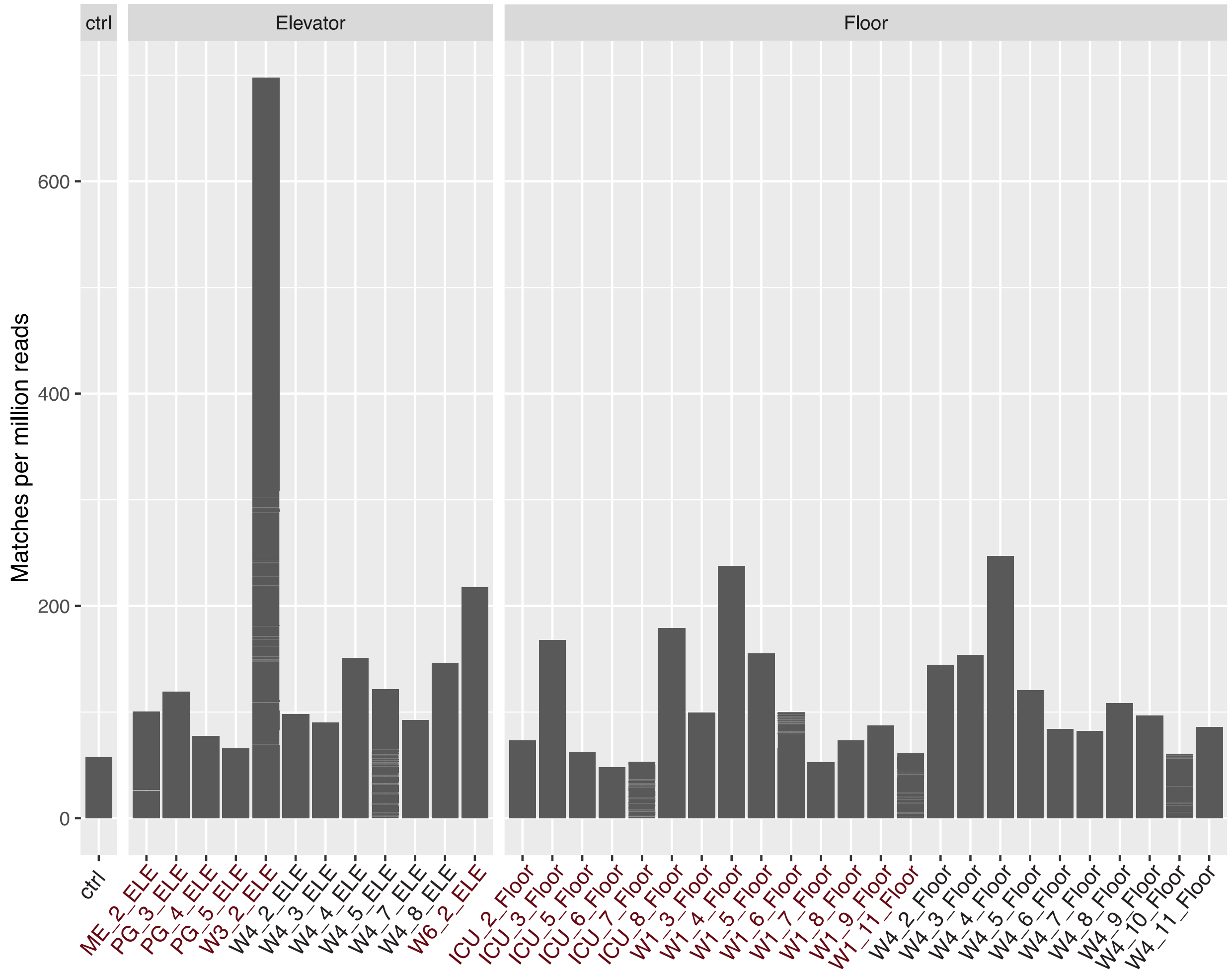

Supplemental Figure G: Reads per million assigned to virulence factor genes based on mapping to the Virulence Factor Database (VFDB, downloaded July 5, 2023). Sample names follow the convention UNIT_WEEK_SOURCE. SARS-CoV-2 positive samples are colored red.

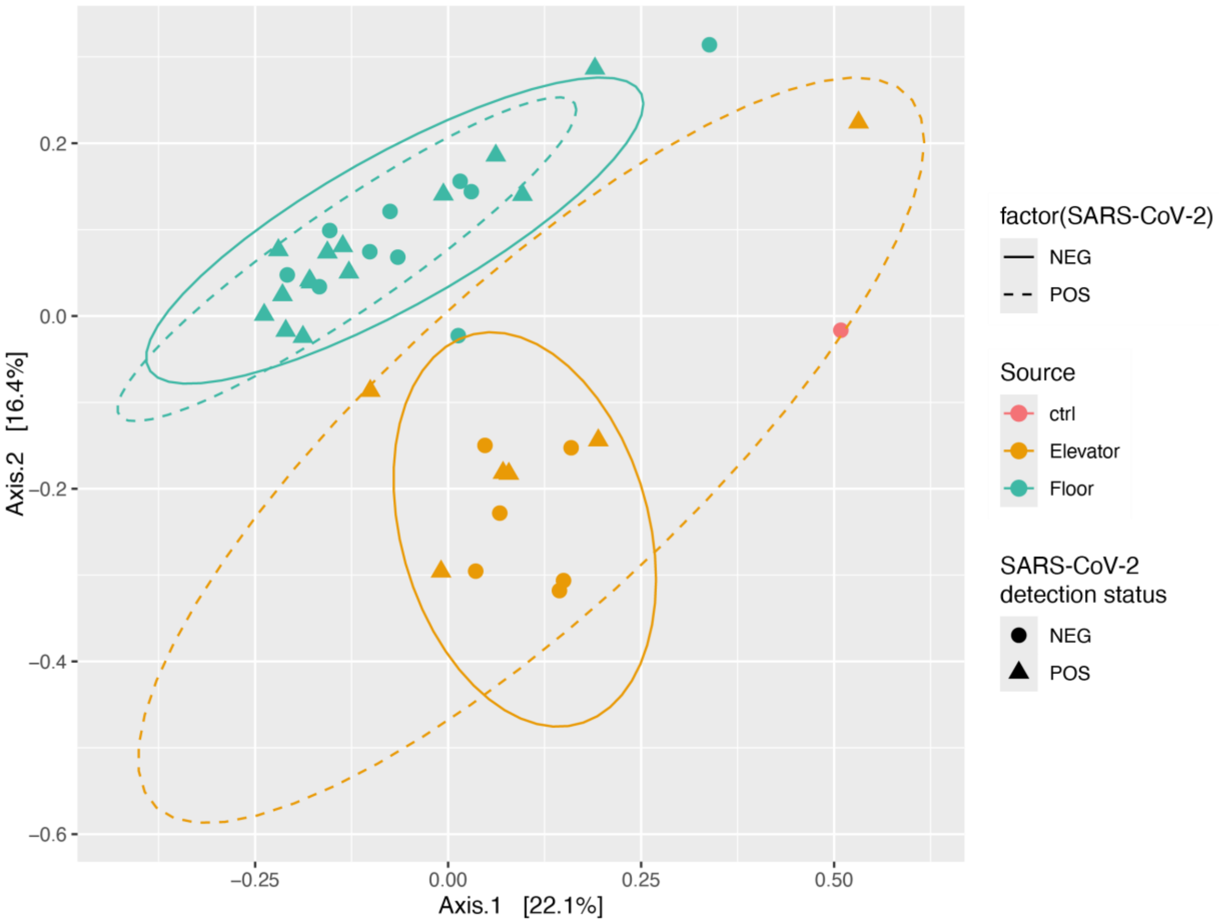

Supplemental Figure H: PCoA ordination of samples based on virulence factor assignments derived from mapping reads to the VFDB (downloaded July 5, 2023). Points are colored by source type (elevator, floor, and control (ctrl)), with SARS-CoV-2 detection indicated by shape. Ellipses encompass SARS-CoV-2 positive (dashed) and negative (solid) samples within each site type.

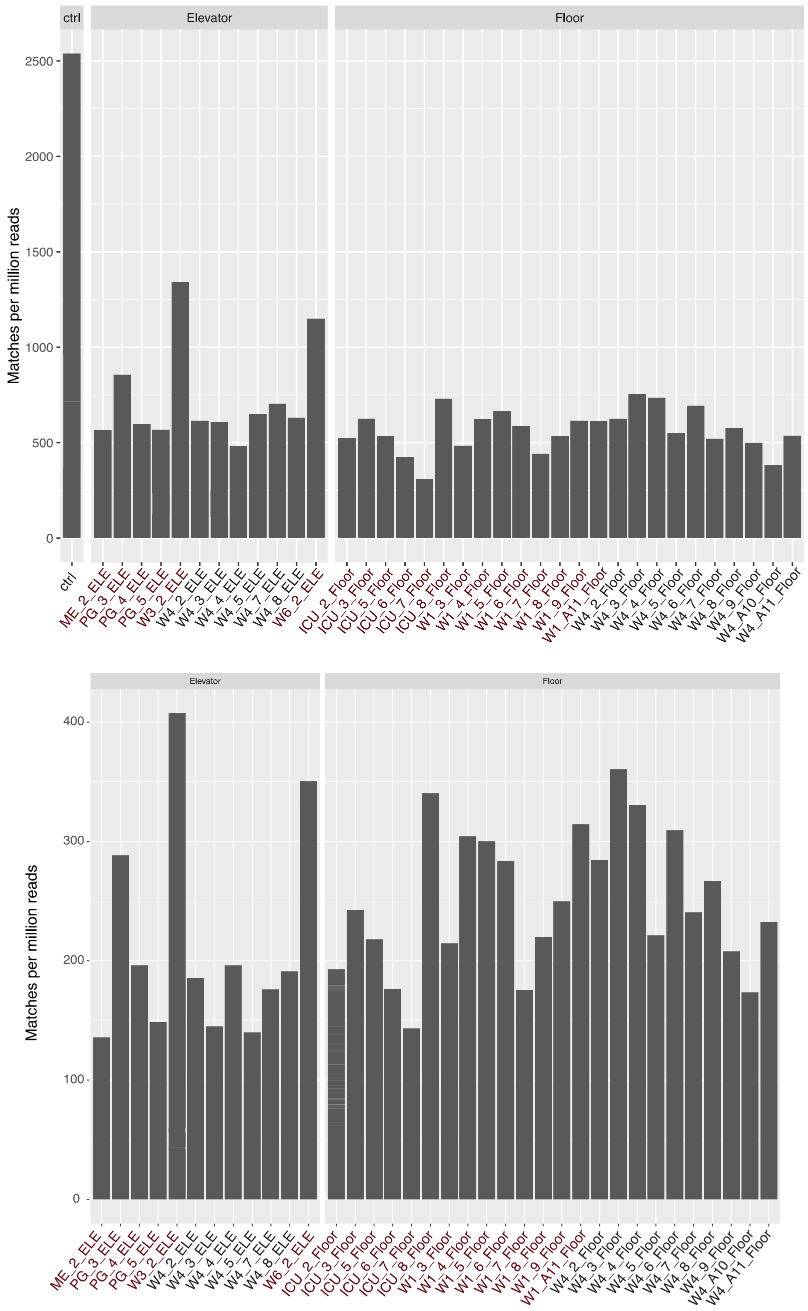

Supplemental Figure I: Reads per million assigned to antimicrobial resistance genes based on mapping to the CARD v3.2.7 and RGI v6.0.0 databases. Sample names follow the convention UNIT_WEEK_SOURCE. SARS-CoV-2 positive samples are colored red. **Top:** raw ARG counts per million reads prior to removal of ARGs identified in the control sample. **Bottom:** ARG counts per million reads following removal of all ARGs identified in the control sample from all samples. Sample names follow the convention UNIT_WEEK_SOURCE. SARS-CoV-2 positive samples are colored red.

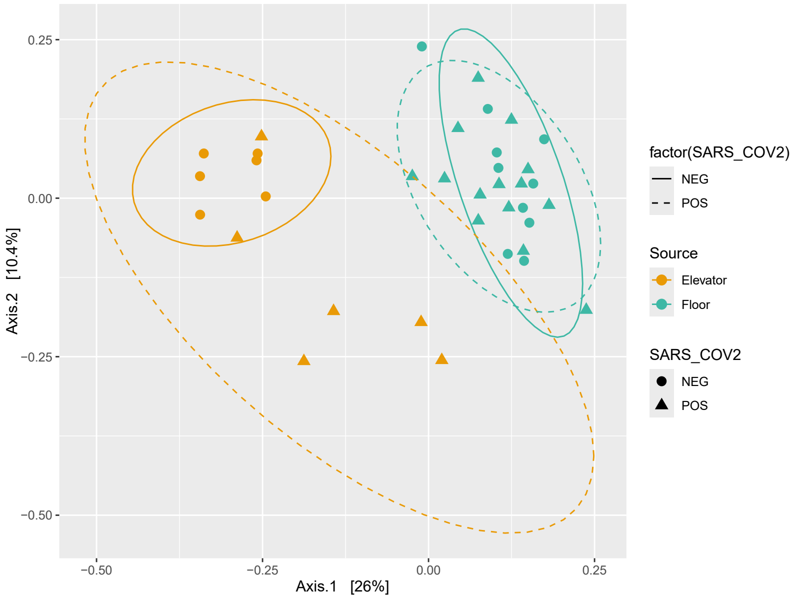

Supplemental Figure J: PCoA ordination of samples based on antimicrobial resistance profiles derived from mapping to the CARD database after removal of control-associated ARGs. Points are colored by source type (elevator, floor), with SARS-CoV-2 detection indicated by shape. Ellipses encompass SARS-CoV-2 positive (dashed) and negative (solid) samples within each site type.

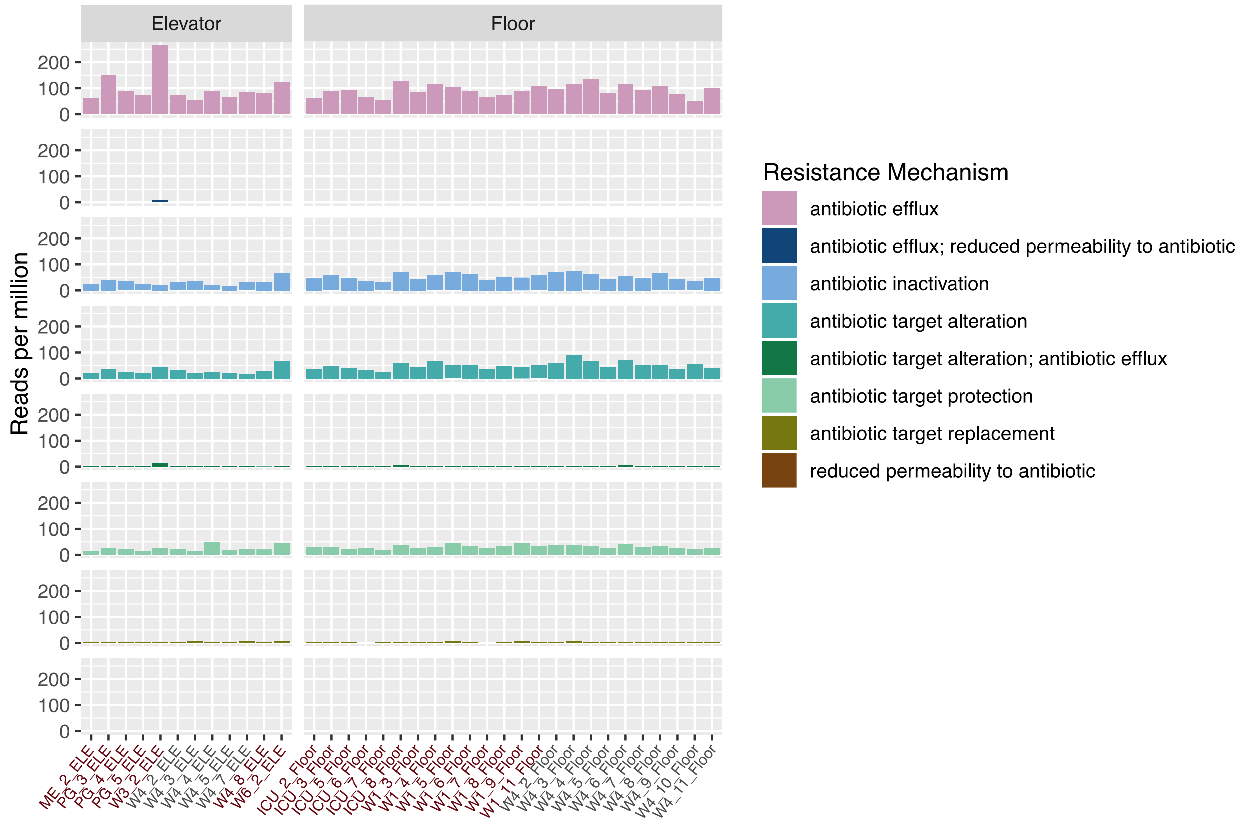

Supplemental Figure K: Bar plot of CARD Resistance Mechanism categorical abundance by read count per million reads faceted by sample Source. Sample names follow the convention UNIT_WEEK_SOURCE. SARS-CoV-2 positive samples are colored red.

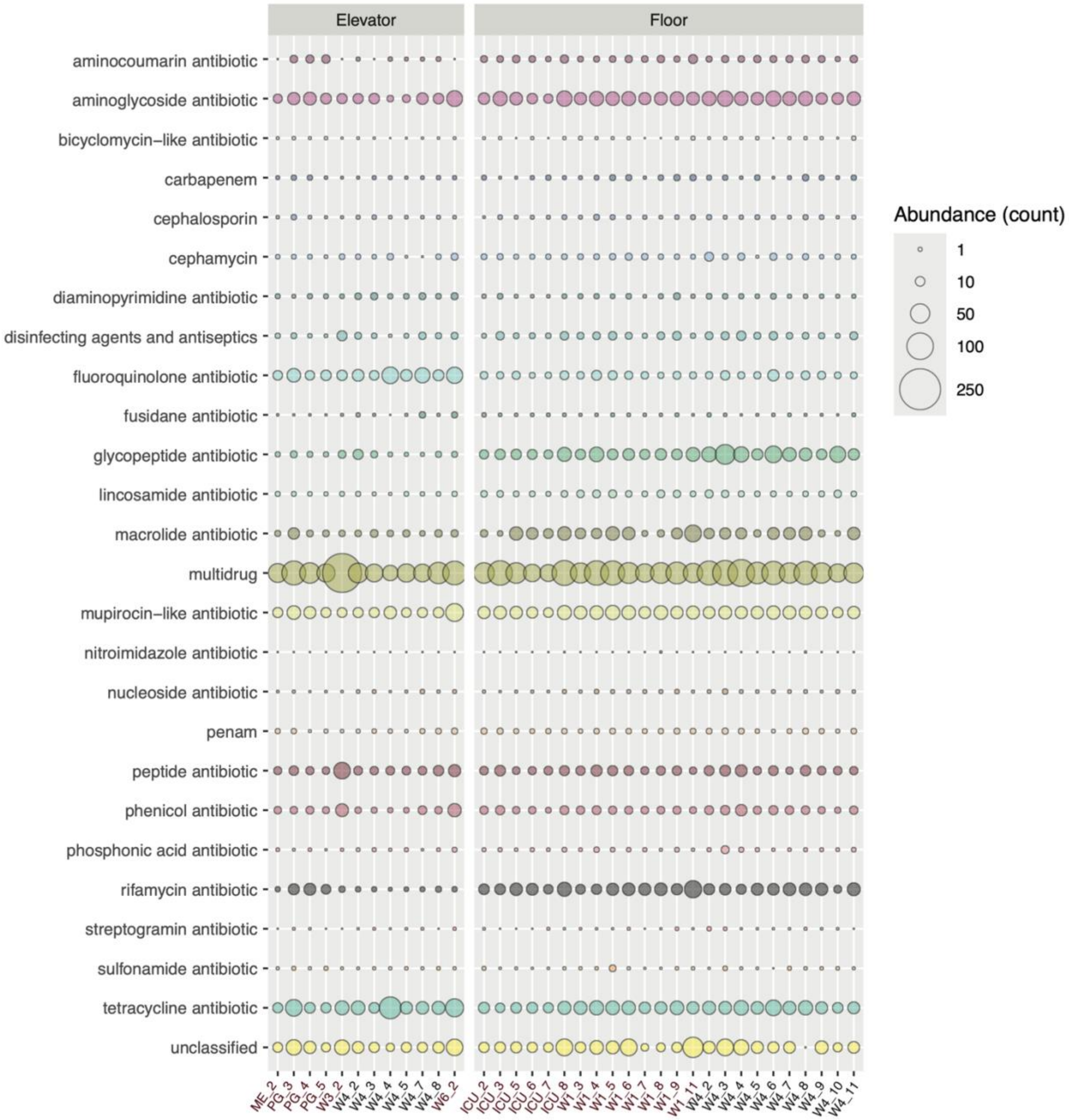

Supplemental Figure L: Bubble plot of CARD Drug Class categorical abundance by read count per million reads, faceted by sample Source. Multidrug is the summed abundance per million reads for all assignations that include resistance to more than one drug class. Sample names follow the convention UNIT_WEEK_SOURCE. SARS-CoV-2 positive samples are colored red.

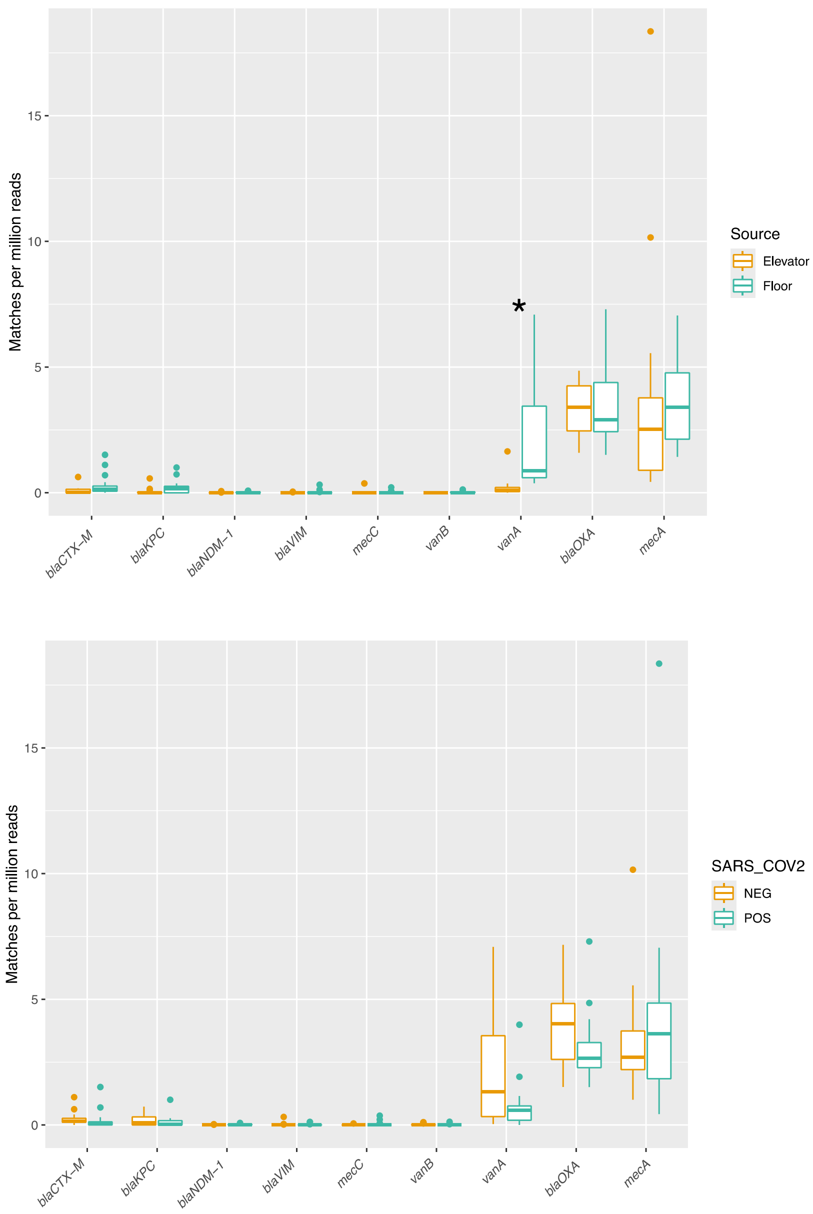

Supplemental Figure M: Clinically relevant ARG prevalences compared across (top) sample Source and (B) SARS-CoV-2 detection status. An asterisk marks the only significant comparison ($p < 0.01$) based on a Kruskal-Wallis test (Supplemental Table B).
